# Thoracic fluid content as a novel and rapid diagnostic indicator of secondary capillary leak syndrome in pediatric patients post-cardiopulmonary bypass

**DOI:** 10.3389/fped.2025.1494533

**Published:** 2025-03-25

**Authors:** Junming Huo, Jie Cheng, Chengjun Liu, Yueqiang Fu, Feng Xu, Jing Li

**Affiliations:** ^1^ Department of Critical Care Medicine, National Clinical Research Center for Child Health and Disorders, Ministry of Education Key Laboratory of Child Development and Disorders, China International Science and Technology Cooperation Base of Child Development and Critical Disorders, Chongqing Key Laboratory of Pediatrics, Children’s Hospital of Chongqing Medical University, Chongqing, China; ^2^ Department of Emergency, National Clinical Research Center for Child Health and Disorders, Ministry of Education Key Laboratory of Child Development and Disorders, Chongqing Key Laboratory of Pediatrics, Children’s Hospital of Chongqing Medical University, Chongqing, China

**Keywords:** thoracic fluid content (TFC), capillary leak syndrome (CLS), indicator, cardiopulmonary bypass (CPB), children

## Abstract

**Objective:**

Capillary leak syndrome (CLS) is an urgent problem in postoperative patients, is challenging to diagnose early, and has a poor prognosis. We investigated a quick and convenient diagnostic indicator of secondary CLS in children after cardiopulmonary bypass (CPB).

**Methods:**

We conducted this single-center, observational, prospective study in the Department of Critical Care Medicine at the Children's Hospital of Chongqing Medical University. All the data were collected within 24 h after cardiopulmonary bypass (CPB). The secondary CLS risk factors were determined using univariate and multivariate logistic regression analysis, and the cut-off point of secondary CLS was found by receiver operating characteristic (ROC) analysis.

**Results:**

Our study included two hundred four pediatric patients in the PICU after cardiopulmonary bypass (CPB). 42.65% (87/204) of patients were diagnosed with secondary CLS. The incidence of acute kidney injury (AKI) was 36.76% (75/204), and the mortality was 5.39% (11/204). Logistic analysis indicated that a pulmonary exudation on chest radiograph, a high thoracic fluid content (TFC) and a higher vasoactive inotropic score (VIS) were independent risk factors for secondary CLS [odds ratio [OR] 23.62, 95% confidence interval [CI] 7.20–90.41, *p* < 0.001; OR 1.08, 95% CI 1.02–1.16, *p* = 0.010; OR 1.06, 95% CI 1.01–1.14, *p* = 0.049; respectively]. According to the ROC analysis, the cut-off point for the TFC was 52 (Ω^−1^).

**Conclusions:**

The TFC plays a key role in the early prediction of secondary CLS in children after CPB, and this novel indicator may help clinicians initiate intensive treatment as early as possible.

## Introduction

China has the most individuals with congenital heart disease (CHD) in the world (1), and the CHD birth incidence has increased from 0.201‰–4.905‰ in the past few decades (2). The proportion of CHD-related deaths in children has risen in recent years (1), and early cardiac surgery may be an opportunity to help these children with CHD survive (3). However, early cardiac surgery may also be associated with increased mortality because of the prolonged inflammation (4). The CLS often occurs in critically ill patients and may lead to multiple organ dysfunction syndrome (5), especially in those children who underwent CPB (4). The CLS has an uncertain incidence and high mortality rate (6). The reported mortality of CLS ranges from 20%–30% (6). Although CLS is common in critically ill patients, uniform diagnostic criteria still need to be established (7), which may inhibit the early recognition of CLS. Wollborn et al. (7) established a scoring system with seven variables to identify CLS patients, but this system may be too cumbersome. A convenient indicator may contribute to the early diagnosis of CLS and provide more intensive treatment for those critical conditions.

TFC has shown promise in predicting CLS (8, 9), a life-threatening condition characterized by fluid shifts and hemodynamic instability (5). Fluid management is key in developing and treating CLS (5). The thoracic cavity can be considered an inhomogeneous electrical conductor (10). Thoracic fluid content (TFC) is derived from the thoracic electrical base impedance (Ω^−1^). Thoracic electrical base impedance was measured according to the amount of thoracic intravascular and extravascular fluid content. The value of TFC seems to be positively associated with the thoracic fluid volume (11). Capillary leak syndrome (CLS) is a disease characterized by increased capillary permeability, which may lead to fluid leakage into the interstitial space (5). The thoracic fluid content may increase in patients with CLS, and we assumed that TFC may play a crucial role in the early diagnosis of secondary CLS.

TFC was demonstrated to be a potential prognostic indicator in critically ill children (4, 12), but its value in the early diagnosis of secondary CLS was unknown. Our study aimed to evaluate the diagnostic value of the TFC in secondary CLS and determine the optimum cut-off point for the TFC. This study may help clinicians recognize secondary CLS early and provide more intensive therapy.

## Methods

### Study designs and patients

This prospective, observational, cohort study was conducted in the Department of Critical Care Medicine at the Children's Hospital of Chongqing Medical University, a National Clinical Research Center for Child Health and Disorders in China. The sample size was determined based on the method reported in the literature (13). Patients hospitalized in the pediatric intensive care unit (PICU) after cardiopulmonary bypass were enrolled in the study from December 2020–December 2022. The inclusion criteria were as follows: (i) aged younger than 18 years; (ii) admitted to the PICU after surgery; (iii) underwent cardiopulmonary bypass (CPB). The exclusion criteria were as follows: (i) refused to participate in the study; (ii) had pleural effusion or pericardial effusion before surgery. This study was approved by the Ethics Committee of Children's Hospital of Chongqing Medical University. File No. 2021 (39). Informed consent was obtained from the statutory guardians of the children. All methods were performed according to the relevant guidelines and regulations.

### Data collection and definitions

**Figure 1:**
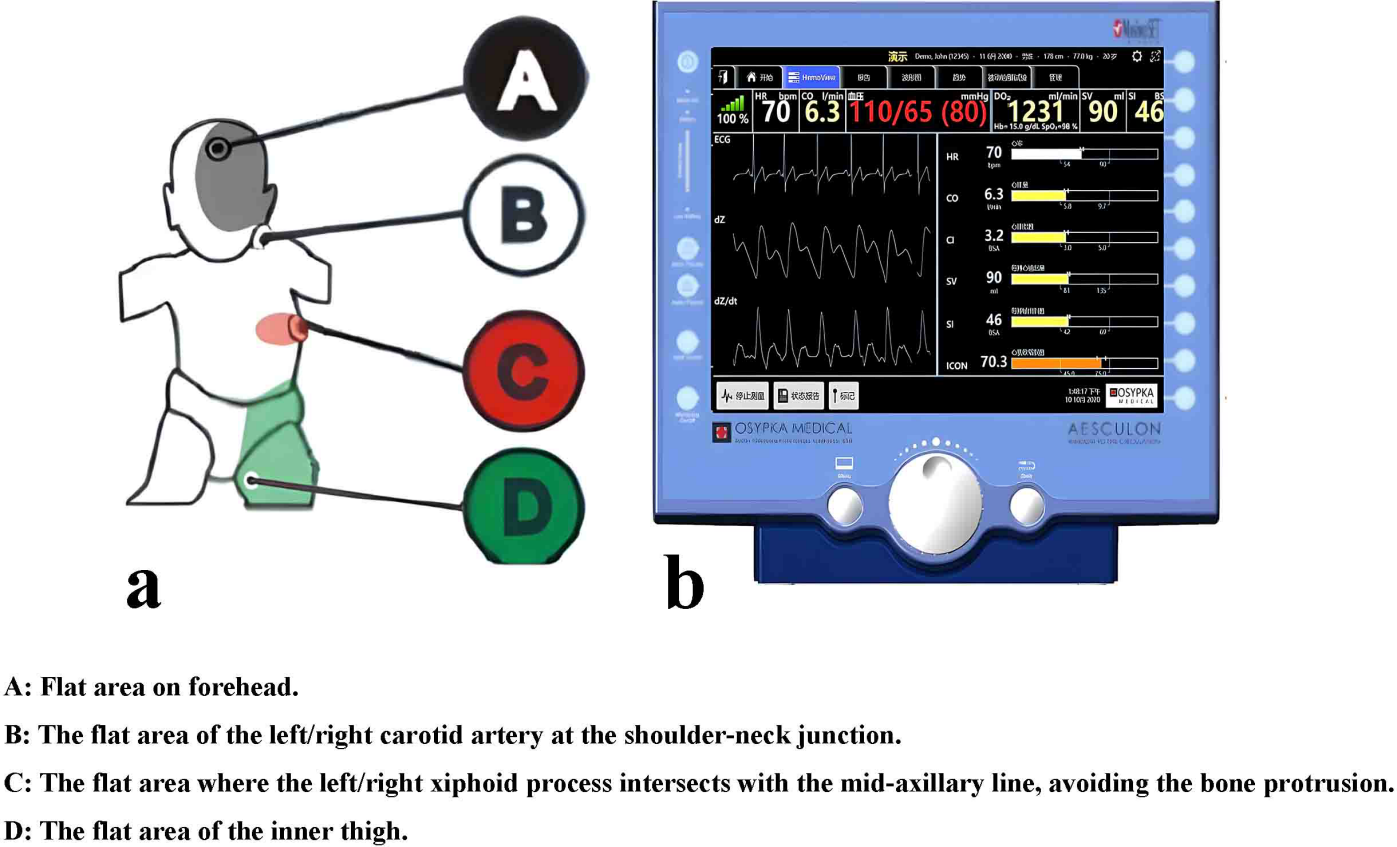
The location of electrodes **(a)** which connected to the ICON monitor **(b)**.

Age, body mass index (BMI), duration of surgery, central venous pressure (CVP), albumin level, intake and output records, chest x-ray results, the incidence of acute kidney injury (AKI), CLS, and mortality were collected from the patient's electronic records. The TFC and stroke volume variation (SVV) were monitored by using a noninvasive AESCULON® device (Osypka Medical, Berlin, Germany. San Diego, California, USA. Website: https://www.osypkamed.com/products/electrical-cardiometry/aesculon/) (14). Four sensors were placed on the left side of the patient's body (Figure 1a). The thorax impedance was measured according to the sensors' low-amplitude, high-frequency electrical current. Based on the impedance, the TFC was determined by using a complex algorithm and detected via the noninvasive hemodynamic monitor (Figure 1b). The risk stratification for congenital heart surgery (RACHS) was used to evaluate the risk of CHD surgery (15). The vasoactive inotropic score (VIS) was used to quantify the degree of hemodynamic support (16). The pediatric sequential organ failure assessment (pSOFA) score was applied to assess the severity of the patients (17). All the data were collected within 24 h after CPB. Secondary CLS was clinically diagnosed by at least two experts with at least 5 years of experience in the intensive care unit and supervised by a senior professor with at least 15 years of experience in the intensive care unit. The discussion among the experts determined the final diagnosis of secondary CLS. They did not know about our study during their clinical diagnosis and treatment. The clinical diagnostic criteria for CLS were as follows (7): (i) positive fluid balance, (ii) hypovolemic shock, (iii) edema with hypoalbuminemia. Furthermore, the AKI was diagnosed according to the Chinese clinical practice guideline for AKI (18). Moreover, hypoalbuminemia was defined as a serum albumin level of less than 3 g/dl (19). The fluid management protocols were refered to Siddall et al. (5).

### Statistical analysis

Continuous variables are medians (quartiles), and categorical variables are frequencies (%). Categorial data were analyzed using the *X*^2^ or Fisher's exact test, and continuous data were analyzed using the Student's *t*-test or the Mann−Whitney *U* test. Variables with *p* values ≤ 0.1 were selected from the univariate logistic regression analysis to the multivariate logistic regression analysis. The receiver operating characteristic (ROC) curve was used to determine the optimum cut-off point for predicting secondary CLS. An AUC more significant than 0.9 indicated a high predictive value (20). We performed the statistical analyses in R software (version 4.3.0). A *p*-level ≤ 0.05 was considered to indicate statistical significance.

## Results

### Study population

**Figure 2:**
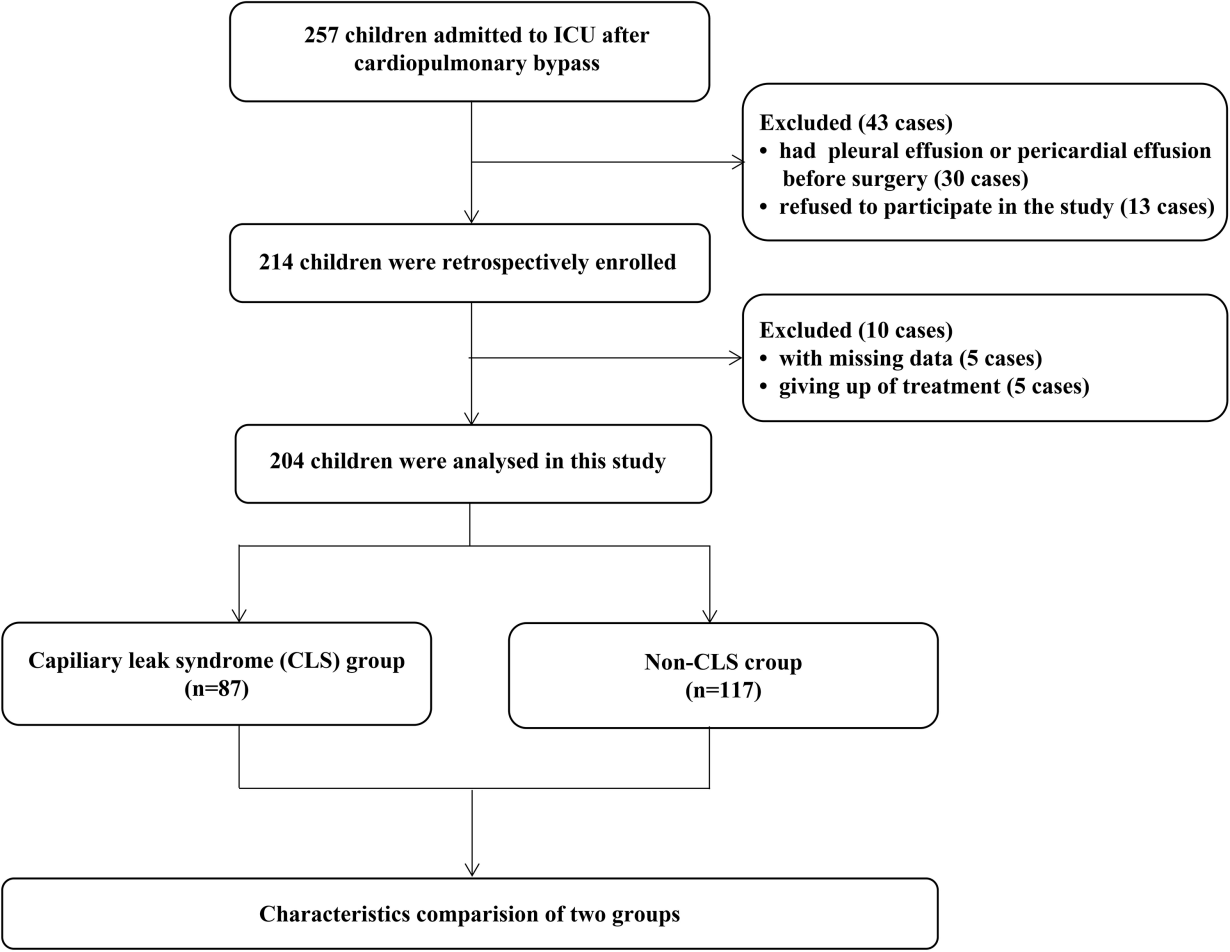
Flow diagram of the population.

From December 2020 to December 2022, 257 children were admitted to the PICU after CPB. In addition, 43 children were excluded at the beginning of the study (30 children had pleural effusion or pericardial effusion before surgery, and 13 children refused to participate). Then, another 10 children were excluded (5 children with missing data and 5 children who gave up the treatment.). Then, another 10 children were excluded (5 children with missing data and five who gave up the treatment). Our study ultimately analyzed 204 children. All the details are shown in Figure 2.

### Clinical characteristics of 204 PICU patients after CPB

The median age was 4.00 (IQR 1.00–11.25) months. The median duration of aortic cross-clamping time (ACT) and CPB were 76.00 (IQR 56.75–98.00) minutes and 112.00 (IQR 94.00–133.00) minutes, respectively. The median TFC was 42.00 (IQR 29.75–64.25) Ω^−1^. Moreover, the median pSOFA score was 6.00 (3.00–12.00). 42.65% (87/204) of patients were diagnosed with CLS. In the first 24 h after CPB, one hundred and forty-nine children had a positive fluid balance, and ninety-four had pulmonary exudation according to chest radiography. The incidence of AKI was 36.76% (75/204), and the mortality was 5.39% (11/204). The details are shown in Table 1.

**Table 1:** Characteristics of 204 pediatric patients in the pediatric intensive care unit (PICU) after cardiopulmonary bypass (CPB).

Characteristics	Number (%)/Median (IQR)
Basic information	
Age (months)	4.00 (1.00–11.25)
Body mass index (BMI) (kg/m^2^)	13.80 (12.10–15.60)
Duration of the surgery	
Aortic cross-clamping time (ACT) (minutes)	76.00 (56.75–98.00)
CPB (minutes)	112.00 (94.00–133.00)
Hemodynamic data	
Stroke volume variation (SVV) (%)	15.50 (11.00–20.00)
Central venous pressure (CVP) (mmHg)	11.00 (8.00–14.00)
Thoracic fluid content (TFC) (Ω^−1^)	42.00 (29.75–64.25)
Postoperative records within 24 h	
Positive fluid balance	149 (73.04%)
Pulmonary exudation on chest radiography	94 (46.08%)
Albumin level (g/L)	36.00 (29.00–40.00)
Scoring systems	
Risk stratification for congenital heart surgery (RACHS)	
1	2 (0.98%)
2	91 (44.61%)
3	99 (48.53%)
4	12 (5.88%)
Vasoactive inotropic score (VIS)	16.00 (10.00–22.00)
Pediatric sequential organ failure assessment (pSOFA) score	6.00 (3.00–12.00)
Outcomes	
Acute kidney injury (AKI)	75 (36.76%)
Capillary leak syndrome (CLS)	87 (42.65%)
Mortality	11 (5.39%)

### Comparisons of clinical characteristics between the CLS and the non-CLS groups

The children diagnosed with CLS had a significantly greater value of TFC than those not diagnosed with CLS [87.00 (IQR 66.50–95.00) vs*.* 41.00 (IQR 29.00–62.00), *p* < 0.001]. The patients with CLS had markedly lower albumin levels than did the patients without CLS [29.00 (IQR 22.50–31.50) *vs.* 37.00 (IQR 29.00–40.00), *p* = 0.001]. Compared with those in the non-CLS group, significantly more patients in the CLS group had a positive fluid balance or pulmonary exudation on chest radiography within 24 h after CPB (95.40% vs*.* 56.41%, *p* < 0.001; 91.95% *vs.* 11.97%, *p* < 0.001; respectively). The values of VIS and pSOFA scores in the CLS group were markedly greater than those in the non-CLS group (24.00 (IQR 20.50–39.00) vs. 15.00 (IQR 8.00–21.00), *p* = 0.002; 20.00 (IQR 18.50–21.00) vs. 5.00 (IQR 3.00–11.00), *p* < 0.001; respectively). Patients with CLS had a significantly greater incidence of AKI and mortality than those without CLS (85.06% *vs.* 0.85%, *p* < 0.001; 12.64% vs*.* 0.00%, *p* < 0.001; respectively). Age, duration of the surgery, SVV, CVP, and the risk stratification for congenital heart surgery were not significantly different between the CLS and the non-CLS groups. The results are presented in Table 2.

**Table 2:** Comparisons of clinical characteristics in CLS and non-CLS group in 204 pediatric patients in the PICU after cardiopulmonary bypass.

Characteristics	Non-CLS group	CLS group	*P*-value
	(*n* = 117)	(*n* = 87)	
Basic information			
Age (months)	4.00 (1.00–12.00)	3.00 (0.70–5.00)	0.320
BMI (kg/m^2^)	13.90 (12.10–15.80)	12.20 (10.95–14.05)	0.061
Duration of the surgery			
ACT (minutes)	76.00 (56.00–98.00)	76.00 (62.50–116.50)	0.308
CPB (minutes)	112.00 (94.00–133.00)	126.00 (106.50–154.50)	0.129
Hemodynamic data			
SVV (%)	15.00 (11.00–20.00)	17.00 (10.50–26.50)	0.480
CVP (mmHg)	11.00 (8.00–13.00)	12.00 (10.50–15.50)	0.247
TFC (Ω^−1^)	41.00 (29.00–62.00)	87.00 (66.50–95.00)	<0.001*
Postoperative records within 24 h			
Positive fluid balance	66 (56.41%)	83 (95.40%)	<0.001*
Pulmonary exudation on chest radiography	14 (11.97%)	80 (91.95%)	<0.001*
Albumin level (g/L)	37.00 (29.00–40.00)	29.00 (22.50–31.50)	0.001*
Scoring systems			
RACHS			0.712
1	2 (1.04%)	0 (0.00%)	
2	87 (45.08%)	4 (36.36%)	
3	92 (47.67%)	7 (63.64%)	
4	12 (6.22%)	0 (0.00%)	
VIS	15.00 (8.00–21.00)	24.00 (20.50–39.00)	0.002*
pSOFA score	5.00 (3.00–11.00)	20.00 (18.50–21.00)	<0.001*
AKI	1 (0.85%)	74 (85.06%)	<0.001*
Mortality	0 (0.00%)	11 (12.64%)	<0.001*

* With statistical significance, *p* < 0.05.

### Risk factors for secondary CLS

Table 3 shows the results of univariate and multivariate logistic regression analyses of risk factors for CLS. According to univariate logistic analysis, pulmonary exudation on chest radiography within 24 h after CPB and a higher value of VIS or pSOFA score, a lower albumin level, and a greater TFC were positively correlated with the incidence of secondary CLS (*p* < 0.001). According to our multivariate logistic analysis, pulmonary exudation on chest radiography within 24 h after CPB, and a higher value of TFC or VIS were found to be independent risk factors for secondary CLS (OR 23.62, 95% CI 7.20–90.41, *p* < 0.001; OR 1.08, 95% CI 1.02–1.16, *p* = 0.010; OR 1.06, 95% CI 1.01–1.14, *p* = 0.049).

**Table 3:** Logistic regression analysis of risk factors for CLS among 204 pediatric patients in the PICU after cardiopulmonary bypass.

Variables	Univariate analysis			Multivariate analysis		
	OR	95% CI	*P*	OR	95% CI	*P*
Pulmonary exudation on chest radiography	84.06	34.46–235.71	<0.001*	23.62	7.20–90.41	<0.001*
TFC (Ω^−1^)	1.13	1.09–1.16	<0.001*	1.08	1.02–1.16	0.010*
VIS	1.09	1.06–1.14	<0.001*	1.06	1.01–1.14	0.049*
pSOFA score	1.49	1.35–1.66	<0.001*			
BMI (kg/m^2^)	0.90	0.81–1.00	0.053			
Albumin level (g/L)	0.76	0.70–0.82	<0.001*			

* With statistical significance, *p* < 0.05.

### ROC curve of the TFC for predicting secondary CLS

**Figure 3:**
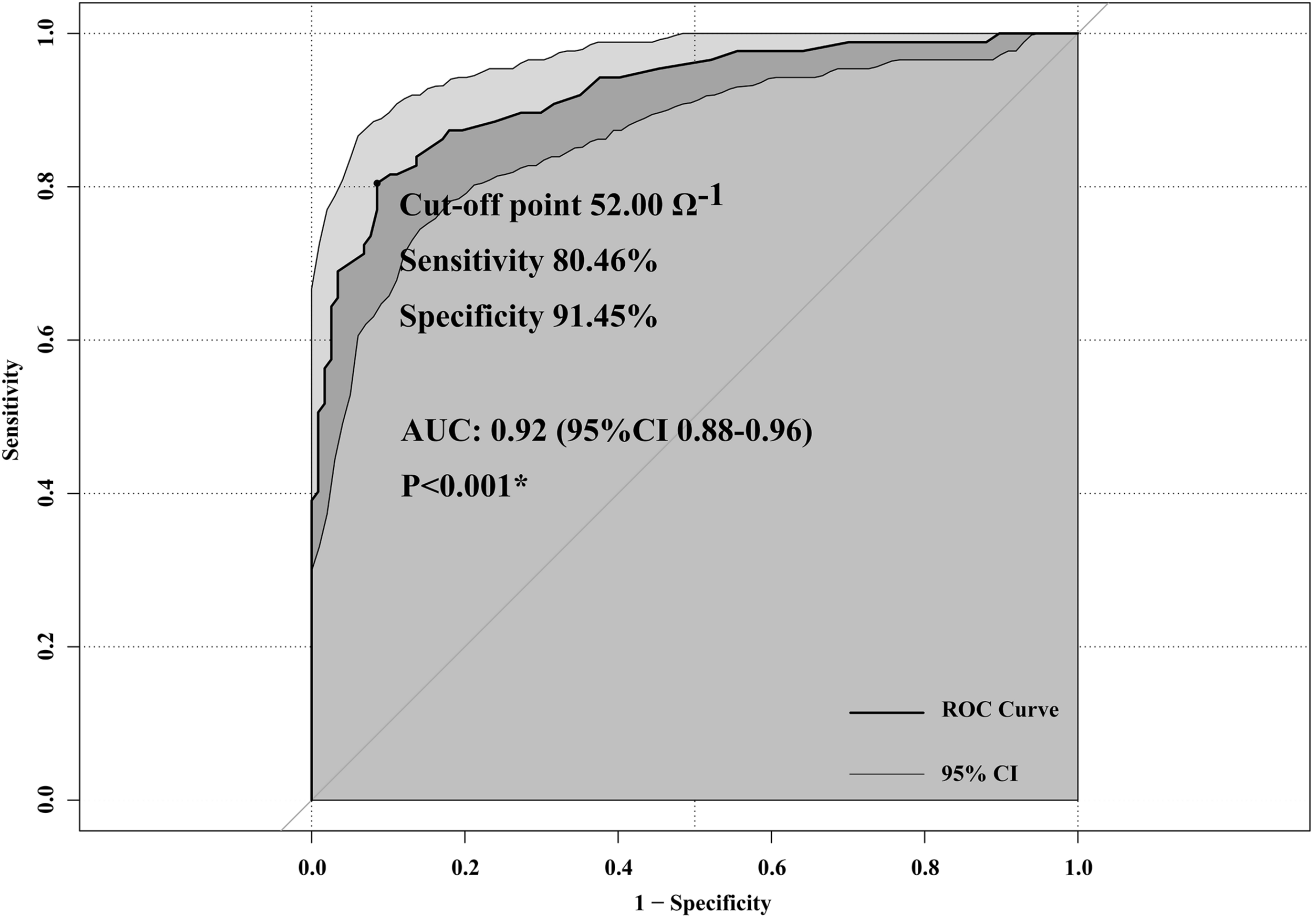
Receiver operating characteristic (ROC) curves of thoracic fluid content (TFC) to predict capillary leak syndrome (CLS).

The ROC curve of the TFC was plotted according to the secondary CLS in Figure 3. The area under the ROC curve (AUC) was 0.92 (95% CI 0.88–0.96), indicating good predictive efficacy. The optimal cut-off point for the TFC was 52.00 Ω^−1^ according to Youden's index methodology, with 80.46% sensitivity and 91.45% specificity. After CPB, patients with a TFC > 52.00 Ω^−1^ were at high risk for CLS.

## Discussion

The secondary CLS can be understood as a result of fluid imbalance and inflammatory responses triggered by surgery. The pathophysiological mechanisms may be as follows (21). First, the surgical trauma and CPB trigger a systemic inflammatory response, releasing cytokines (e.g., IL-6, TNF-α) that disrupt endothelial integrity, increasing capillary permeability. Second, CPB-induced ischemia-reperfusion injury and oxidative stress damage endothelial cells, further exacerbating capillary leakage. Third, postoperative fluid management challenges, such as excessive fluid administration, can worsen interstitial edema and organ dysfunction. Last, altered hemodynamics post-CHD surgery, including low cardiac output syndrome, contributes to hypoperfusion and endothelial injury, perpetuating CLS.

Thoracic Fluid Content (TFC) measured via electrical bioimpedance is a non-invasive, cost-effective tool for monitoring fluid status in critically ill children (12). It provides real-time data on thoracic fluid dynamics, aiding in the early detection of conditions like pulmonary edema and fluid overload, which are common in PICU. Its low cost and ease of use make it adaptable to various clinical settings, reducing the need for invasive procedures. Most importantly, it is more convenient than the diagnostic scoring system established by Wollborn et al. (7).

Fathy et al. (22) demonstrated the prognostic value of the TFC for predicting weaning failure in critically ill surgical patients, and Sumbel et al. (12) reported that the TFC can predict outcomes in critically ill children. Our study found that the TFC was an independent risk factor for secondary CLS. As the value of TFC increased, patients had a 1.08-fold more significant risk of CLS. Furthermore, according to the ROC analysis, patients with a value of TFC > 52.00 Ω^−1^ had a risk of CLS, with high predictive ability (AUC 0.92, 95% CI 0.88–0.96, *p* < 0.001). The prognostic value of the TFC for secondary CLS may be explained as follows.

The electrical base impedance of the thoracic tract changes with the amount of fluid and will be detected and quantified as the TFC using a noninvasive AESCULON® device (12, 14). Yan et al. (11) previously reported a positive correlation between TFC and fluid content, and Perko et al. (9) also demonstrated the association between TFC and fluid balance during cardiac surgery. The capillary permeability to protein was increased in patients with CLS, which may lead to hypovolemia and tissue edema (5). The TFC value changes with the thoracic fluid volume (23). Therefore, the TFC value could indirectly measure the capillary permeability to proteins and might help predict CLS's occurrence. Elevated TFC levels correlate with increased extravascular lung water (12), a key feature of CLS (5), enabling early intervention. This predictive capability is particularly valuable in managing high-risk pediatric patients, potentially improving outcomes by facilitating timely therapeutic measures.

Inflammatory storms are believed to be the potential cause of CLS (5). For patients who undergo CHD surgery, the anesthesia, medication, vascular injury, and surgical stress might induce an inflammation response (4). The lung is the most significant and complex immune organ in the human body and is maximally exposed to the proinflammatory cascade (8). It has already been demonstrated that exudative pleural effusions are involved in all causes of CLS (5). Similarly, our study found that the CLS group had significantly more patients with pulmonary exudation on chest radiography than did the non-CLS group (91.95% vs*.* 11.97%, *p* < 0.001). Furthermore, we also found that patients with pulmonary exudation on chest radiography within 24 h after CPB had an approximately 24-fold risk of CLS (OR 23.62, 95% CI 7.20–90.41, *p* < 0.001). The high OR for pulmonary infiltration remained consistent, suggesting that it was not an artifact of model instability but reflected a strong association in our study population. The high OR may be attributed to the specific characteristics of our cohort, where pulmonary exudation was a prominent and severe manifestation of CLS (5). This finding emphasizes the key role of pulmonary involvement in the pathophysiology of CLS. However, the generalizability of these results may be limited by the specific characteristics of our study population, such as disease severity and demographic composition. Further validation in larger, more diverse cohorts is essential to confirm these observations and explore potential variations across different clinical settings. The perioperative patients were routinely administered with inotropes or vasopressors. Patients with CLS suffer from hypovolemic shock (7). VIS was used to evaluate the degree of cardiovascular support in clinical trials, and a higher VIS might be correlated with poor prognosis (16). Our study also demonstrated that a higher value of VIS was correlated with a greater risk of CLS (OR 1.06, 95% CI 1.01–1.14, *p* = 0.049).

Patients with CLS always exhibit a loss of protein-rich fluid from the intravascular space to the interstitial space, which may lead to hypoalbuminemia and hypovolemia (5). Both fluid overload and hypovolemia can cause organ injury. Fluid management is crucial for critical patients (24) and patients with CLS (5). However, in our study, a positive fluid balance within 24 h and an albumin level were not found to be independent risk factors for CLS. We assumed this difference may be attributed to the different treatments between the two groups of children after cardiac surgery, such as balance management and colloid supplementation (such as albumin). The group without CLS received routine monitoring and treatment after surgery, while the group with CLS received diuresis, colloid supplementation (such as albumin), peritoneal dialysis, and even blood purification treatment. Furthermore, there is no consensus on defining fluid overload (24), which may also lead to difficulty evaluating the association between fluid management and prognosis.

Our study still has some limitations. First, this was a single-center study with a relatively small sample size. Sample selection bias, insufficient statistical power, and the differences between different levels of medical institutions may limit the external validity of this study. However, this study was conducted at the National Clinical Research Center for Child Health and Disorders in China, which admitted patients from all over Southwest China, and this may help to improve the persuasiveness of our results. Furthermore, a multicenter study may be needed. Second, our study enrolled only children. Therefore, our results are inapplicable to adults. Third, there are no consensus diagnostic criteria for CLS, which may limit the extrapolation of our data to other centers. In our study, CLS was diagnosed by at least two experienced clinicians and supervised by a senior professor. The diagnostic criteria were established according to the pathophysiology of CLS (7, 12). Uniform diagnostic criteria for CLS are expected to be established in the future.

## Conclusions

TFC may serve as a novel diagnostic indicator of secondary CLS in PICU children after cardiopulmonary bypass, and patients with a TFC > 52.00 Ω^−1^ are at high risk of CLS.

## Data Availability

The original contributions presented in the study are included in the article/Supplementary Material, further inquiries can be directed to the corresponding author.
